# Editorial: Promising strategies for vaccine messages: The message and the source

**DOI:** 10.3389/fpubh.2022.971283

**Published:** 2022-08-31

**Authors:** Bridget J. Kelly, Amy E. Leader, Nora B. Henrikson

**Affiliations:** ^1^Science in the Public Sphere Program, Center for Communication Science, RTI International, Durham, NC, United States; ^2^Division of Population Science, Department of Medical Oncology, Thomas Jefferson University, Philadelphia, PA, United States; ^3^Kaiser Permanente Washington Health Research Institute, Seattle, WA, United States

**Keywords:** vaccine hesitancy, message tailoring, trusted information source, outbreak communication, immunization

Two years into the COVID-19 pandemic, countries around the world continue to struggle with low vaccination rates. As of June 13, 2022, only 68% of the world's population had received at least one dose of the COVID-19 vaccine (1). While access and other structural barriers explain part of the gap, vaccine hesitancy also plays a role. Vaccine hesitancy has been an important public health issue in recent decades, but has never received as much attention as it has since the spring of 2020 with the start of the COVID-19 pandemic. Despite countless research studies on the topic, questions remain about how best to design vaccine messages and health communication campaigns that will be influential in promoting behavioral intentions.

The literature is clear that one-size-fits-all messaging approaches are not effective (2). The numerous systematic reviews on vaccination published since the start of the pandemic have identified common themes like the importance of trusted sources and context-specific barriers and facilitators (3, 4). These papers are consistent with longstanding public health theory and literature suggesting message strategies should be tailored to the population of interest to address the beliefs, norms, barriers and other factors most likely to influence behavioral intentions (5–7). The papers in this special issue underscore these findings.

The objective of this special collection entitled, “Promising strategies for vaccine promotion: The message and the source,” was to bring together recent research studies from around the world that have explored questions related to health communication and vaccine promotion. The resulting collection of five papers includes four observational studies and one case study. Specifically, the collection includes a retrospective piece on collaborating with two nutrition-focused, U.S.-based community organizations to deliver vaccine messaging (Rauh et al.); a qualitative interview study of students in China on COVID-19 vaccination (Luo and Song); a survey of medical students in China related to HPV vaccine hesitancy (Zhou et al.); a survey of college students in India to assess COVID-19 vaccination intentions (Jain et al.); and a social network and sentiment analysis of social media posts (Gao et al.). As diverse in methods as these five papers are, several overlapping themes emerged among them.

The first was specific to the content of the messages themselves: vaccine safety and efficacy were top of mind in all study populations. In the survey of more than 700 medical students by Zhou et al., a large percentage were concerned about the safety of HPV vaccines. Interestingly, a majority of the students were unsure where to obtain reliable information about the HPV vaccine, despite their daily immersion in medical literature and frequent engagement with scientific sources. In 55 interviews with young Chinese students, Luo and Song found that vaccine information insufficiency augmented other barriers to vaccine intention. Some of the interview participants shared that more detailed information comparing the Chinese domestic vaccines to vaccines available internationally might have alleviated their concerns and shifted their intentions to vaccinate. Both studies illustrate that the public is often uncertain about the ingredients in vaccines and how people react to them. Findings that safety, efficacy (8) and information insufficiency more broadly (9), are important predictors are consistent with other recent research. In an era in which transparency is paramount, the public health community is wise to continue to educate its constituents about vaccine creation, production, distribution, and outcomes to reduce vaccine hesitancy.

A second theme that emerged was the need to tailor messaging to the specific population and context. Luo and Song identified barriers specific to Chinese young adults—Ti Zhi (the individual constitution) and beliefs that vaccines' advantages are weak related to non-medical prevention measures. These beliefs have not been frequently cited elsewhere. Jain et al. found that in India, trust in the domestic vaccine was high, which they contrasted with research from other parts of the world. Both findings highlight the importance of assessing motivators to vaccinate in specific populations (5).

The third theme was the importance of trust in the message source. The analysis by Gao et al. revealed more positive sentiment and less focus on concerns about vaccine safety following official announcement by the Chinese government, suggesting that once officials had endorsed the vaccines, people were more willing to be vaccinated. Zhou et al. found those who obtained information from a doctor (as opposed to another source) had lower rates of vaccine hesitancy. Similarly, Jain et al. found that trust in the healthcare system was a significant predictor of vaccine uptake among college students in India. Prior research has shown countries with higher trust in government have lower infection rates and higher vaccine acceptance (10, 11). For countries like the U.S., where survey research shows trust in government has declined over time (12), future work should consider strategies for increasing that trust.

Rauh et al. put the idea of trusted source into practice. The authors, like, Jain et al., identified the potential for peer-to-peer communication via vaccine ambassadors as a promising strategy in reaching pregnant and postpartum community members. Rauh et al. also demonstrated how trusted community-based organizations can become valuable vaccine messengers, even when healthcare is not their primary mission. Training trusted messengers to deliver accurate vaccine safety and efficacy information, in lay language, may be a practical way to overcome vaccine hesitancy in communities that are doubting or questioning vaccines. Partnering with community organizations and lay health workers to deliver tailored interventions is the cornerstone of public health practice.

We should mention limitations of the collection of studies. While these observational studies can provide insights into the types of messages that may be needed, without randomized trials or intervention studies, we cannot make definitive claims about the types of messages or sources that are most effective in delivering vaccine information.

Audiences are heterogeneous and have varied informational needs. As a result, formative research to determine which messages and sources resonate with a particular audience, and tailoring messages to those findings may ensure higher likelihood of success.

## Author contributions

BK: collaborated on outline, wrote the first draft of the editorial, and revised based on co-authors' feedback. AL: collaborated on outline, revised multiple drafts, and provided additional feedback. NH: revised multiple drafts and provided citations and other feedback. All authors contributed to the article and approved the submitted version.

## Conflict of interest

The authors declare that the research was conducted in the absence of any commercial or financial relationships that could be construed as a potential conflict of interest.

## Publisher's note

All claims expressed in this article are solely those of the authors and do not necessarily represent those of their affiliated organizations, or those of the publisher, the editors and the reviewers. Any product that may be evaluated in this article, or claim that may be made by its manufacturer, is not guaranteed or endorsed by the publisher.
